# Effect of myocyte-fibroblast coupling on the onset of pathological dynamics in a model of ventricular tissue

**DOI:** 10.1038/srep40985

**Published:** 2017-01-20

**Authors:** S. Sridhar, Nele Vandersickel, Alexander V. Panfilov

**Affiliations:** 1 Department of Physics and Astronomy, Ghent University, Ghent, Belgium; 2 Moscow Institute of Physics and Technology (State University), Dolgoprudny, Moscow Region, Russia

## Abstract

Managing lethal cardiac arrhythmias is one of the biggest challenges in modern cardiology, and hence it is very important to understand the factors underlying such arrhythmias. While early afterdepolarizations (EAD) of cardiac cells is known to be one such arrhythmogenic factor, the mechanisms underlying the emergence of tissue level arrhythmias from cellular level EADs is not fully understood. Another known arrhythmogenic condition is fibrosis of cardiac tissue that occurs both due to aging and in many types of heart diseases. In this paper we describe the results of a systematic in-silico study, using the TNNP model of human cardiac cells and MacCannell model for (myo)fibroblasts, on the possible effects of diffuse fibrosis on arrhythmias occurring via EADs. We find that depending on the resting potential of fibroblasts (*V*_*FR*_), M-F coupling can either increase or decrease the region of parameters showing EADs. Fibrosis increases the probability of occurrence of arrhythmias after a single focal stimulation and this effect increases with the strength of the M-F coupling. While in our simulations, arrhythmias occur due to fibrosis induced ectopic activity, we do not observe any specific fibrotic pattern that promotes the occurrence of these ectopic sources.

Mechanical contraction of the heart is preceded by the propagation of electrical waves of excitation. Under pathological conditions, regular wave propagation can breakdown creating reentrant or chaotic patterns of electrical activity^1^ known as arrhythmias. Such arrhythmias manifest as assynchronous or irregular contraction of the heart muscles which, if untreated, can result in the cessation of blood flow followed by sudden cardiac death^2^. In fact, cardiac arrhythmias are one of the leading killers in the industrialised world^3^. While our understanding of the mechanisms underlying the onset of arrhythmia remain incomplete, several physiological factors that increase the vulnerability of cardiac tissue to arrhythmia have been identified^4^,^5^. One of them is the presence of abnormal action potentials in cardiac cells called *early afterdepolarizations* (EADs)^6^, which is essentially the reversal of the action potential before complete cellular repolarization. EADs have been observed in cases of heart failure^7^, long QT syndrome^8^ and are also induced under the action of pharmacological drugs^9^ or conditions of oxidative stress^10^,^11^. Even though there has been significant improvement in our understanding since the earliest observations of EADs nearly half century ago^12^, there still remain several unanswered questions about the mechanisms linking EADs and arrhythmias. EADs occur in cells whose ability to completely repolarize is significantly reduced. This can happen when the net outward current required to repolarize the cardiac cell is not sufficient, either due to an excess of inward current or reduced outward current or both. Thus, at the level of the cell the occurrence of EAD is linked with ionic conductances and gating variables. At the tissue level, the generation of EADs has been shown to be a chaotic process that synchronizes spatially to produce waves that can overcome source-sink mismatches^6^.

Another well established arrhythmogenic condition is fibrosis of cardiac tissue that occurs as a result of proliferation of fibroblasts. Fibroblasts and myocytes are the two major cell types in mammalian heart muscle tissue, with the former outnumbering the latter even while occupying a much smaller volume of the heart^13^. While myocytes are functionally responsible for the cardiac electrical action, the role of fibroblasts pertain to maintaining the structural and electro-mechanical integrity of the heart^13^ and repair post-injury or disease^14^. In the normal heart, fibroblasts are much smaller than the myocytes and do not influence the electrophysiology of the myocytes. However, in aged hearts, and in many forms of heart diseases the number of fibroblasts can substantially increase (up to 40%)^15^. Further, in injured hearts fibroblasts may differentiate into much larger myofibroblasts. Fibrosis may substantially affect wave propagation in the heart and it is well established that fibrosis creates a substrate for the initiation of ventricular and atrial arrhythmias^16^,^17^. Several studies have reported the existence of heterocellular gap junctions between fibroblasts and myocytes^18^,^19^,^20^. Although the exact nature of myocyte-fibroblast coupling *in vivo* is still controversial^14^, there is substantial evidence for the existence of gap-junctional coupling between myocytes and fibroblasts *in vitro* under both physiological and pathological conditions^21^. Both experiments and simulation studies have shown that coupling between myocytes and fibroblasts significantly alter conduction properties of the cardiac tissue^18^,^22^ and myocyte excitability^23^, induce automaticity^20^, modify action potential duration (APD)^24^,^25^, and depolarize the resting membrane potential^19^,^24^ of myocytes.

Recently, there has been much interest in understanding the proarrhythmic tendencies of fibrosis, especially through its effect on afterdepolarization^26^. These afterpotentials can result in triggered activity causing fibrillation in both the atria^27^ and the ventricles^28^,^29^. Computational studies have shown that at intermediate ranges of coupling between myocytes and fibroblasts EADs can induce ectopic activity^30^ and the possibility of increased ectopic activity in fibrosis has been connected to source-sink mismatch^31^. EAD formation in rabbit myocytes was shown to be promoted by myocyte fibroblast coupling in a dynamic patch clamp experiment^32^. A recent paper^33^ studied the effect of regional heterogeneity and fibroblast-myocyte coupling on the onset of ectopic activity using a model of human heart tissue. The authors described the effect of fibroblast distribution and the coupling strength on ectopic activity for the case where fibroblasts are randomly attached to myocytes. All the papers referred to above provide excellent examples of research performed for particular physiological conditions and at the levels of organization such as a single cell, a small patch of cardiac tissue etc. Generally numerical studies are performed for limited parameter sets (usually two or more). Further, in most of the in-silico examples given above fibroblasts have been considered to be either an inexcitable obstacle or a “passive” RC circuit^30^,^31^,^33^. However, recent patch-clamp experiments have identified the presence of inward rectifying potassium (*K*_*ir*_), time and voltage gated potassium currents (*K*_*iv*_) in ventricular fibroblasts^34^,^35^. The aim of our study is therefore to perform a detailed in-silico study of the effect of fibrosis on EAD related arrhythmias using a human ventricular cell model (TNNP-TP06)^36^,^37^ coupled with a state-of-the-art fibroblast model expressing *K*_*ir*_ and *K*_*iv*_ currents, *viz.* MacCannell “active” fibroblasts^25^. While “active” fibroblast models have been used to study the onset of EADs in single cells^32^ and loading effect on myocytes^24^, to the best of our knowledge our work is the first to describe the effect of inserted “active” fibroblasts on the spatiotemporal dynamics in a 2D medium. In our system the “active” fibroblasts act as heterogeneities in the local ionic properties of the 2D tissue. While electrotonic interaction between myocytes and randomly inserted passive fibroblasts have been shown to increase the vulnerability for reentrant arrhythmia^38^,^39^, in our study we describe the effect of such M-F coupling on the onset of EAD induced focal arrhythmia. We perform a comprehensive study over a wide range of myocyte and fibroblast parameters and describe excitation patterns with EAD and fibrillatory wave dynamics similar to those identified in the absence of fibrosis by Vandersickel *et al*.^40^. We also study the effect of M-F coupling (by varying parameters such as coupling strength, fibroblast capacitance and resting membrane potential) on the excitation patterns and the likelihood of their occurrence. We find that depending on the value of the fibroblast resting potential, the coupling can have opposite effects on the myocyte dynamics: A less negative fibroblast resting membrane potential promotes EADs, while a more negative value of resting membrane potential suppresses such EADs. However it should be noted that the M-F coupling does not change the type of spatial EAD patterns. More importantly, irrespective of the parameter values M-F coupling always promotes the occurrence of pathological dynamics such as spiral wave fibrillation.

## Methods

We will first describe the cell models and the parameter regimes that are considered in this paper. To describe the ionic activity of a myocyte we use the TNNP-TP06 model of human ventricular cell^36^,^37^. The time variation of the transmembrane voltage *V* for myocytes is given as,


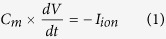


Here *C*_*m*_ is the cell capacitance per unit surface area and *I*_*ion*_ is the sum of all ionic currents:


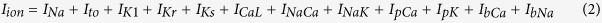


where *I*_*Na*_ is the Sodium current, *I*_*to*_ is the transient outward current, *I*_*K*1_, *I*_*Kr*_ and *I*_*Ks*_ are the inward rectifier, delayed rectifier and slow delayed rectifier Potassium currents, *I*_*CaL*_ is the L-type *Ca*^2+^ current, *I*_*NaK*_ is the *Na*^+^/*K*^+^ pump current, *I*_*NaCa*_ is the *Na*^+^/*Ca*^2+^ exchanger current, *I*_*pCa*_ and *I*_*pK*_ plateau Calcium and Potassium currents and *I*_*bCa*_ and *I*_*bNa*_ are the background *Na*^+^ and *Ca*^2+^ currents. To obtain EADs we have made a few modifications to the L-type Calcium current *I*_*CaL*_ as suggested in literature^40^,^41^. Specifically, the time constant of the f-gate of the L-type Calcium current (*τ*_*f*_) was reduced by half^40^,^41^, resulting in some shortening of the action potential. In order to compensate for this reduction, the value of conductance of the Calcium (*G*_*CaL*_) is increased by a factor of two^40^. In order to draw two-parameter phase portraits we vary *I*_*CaL*_ and *I*_*Kr*_ in myocytes by changing the maximal conductances of *G*_*CaL*_ and *G*_*Kr*_ respectively, since these currents are known to be responsible for the onset of EADs^41^,^42^. The electrophysiological properties for the fibroblasts are described using the MacCannell “active” fibroblast model^25^. The ionic currents in this model include the time- and voltage -dependent Potassium currents *I*_*fKv*_, inward rectifying Potassium current *I*_*fK*1_, *I*_*fNaK*_ a Sodium-Potassium pump current and a background Sodium current *I*_*bNa+*_. The uncoupled resting membrane potential for this model is −49.6 *mV*. In our study we use two sets of values for the fibroblast properties to simulate “fibroblasts” and “myofibroblasts” respectively as suggested in literature^25^,^32^,^34^. For “fibroblasts”, we use a cell capacitance *C*_*F*_ = 6.3 pF and for the larger myofibroblasts, we use a cell capacitance of *C*_*F*_ = 50 pF. The values of fibroblast resting membrane potentials used are *V*_*FR*_ = −49.7 mV and *V*_*FR*_ = −24.5 mV. Different resting membrane potentials were obtained by shifting the gating variable voltage dependence of the time dependent Potassium current^24^. For the gap junctional coupling (Gs), we have chosen values falling in the range that are considered to be representative of the effect of fibroblasts in cell-cultures^24^. The values used for the simulations in the paper are listed in Table. 1.

**Figure 1:**
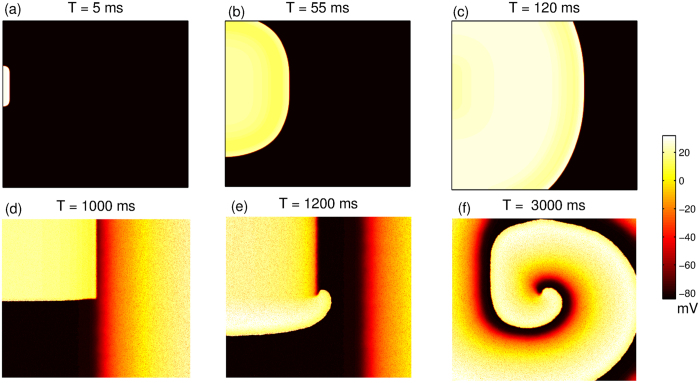
Pseudocolor image of the transmembrane potential V for the two-dimensional system of size 512 × 512 describing protocols P1: Initiating a wavefront from a position slightly off the center of the simulation domain (**a–c**) and P2: Creating a spiral wave via S1–S2 stimulation (**d–f**). Once S1 wave propagates over half the domain, the first quarter of the domain is stimulated (**d**), creating a curved wavefront (**e**) that slowly evolves into a complete spiral wave of excitation (**f**).

For the single cell simulations, the computation is performed using the forward Euler scheme with time- step 0.02 ms. The single cell is stimulated every 500 ms by a stimulus of strength 20 *μA*/*mm*^2^ applied for 2 ms. The 2D simulations have been carried out on a monodomain isotropic cardiac tissue with the systems discretized on a square lattice of size *L* × *L* where *L* = 1024. Each grid point corresponds to a group of myocytes or a cluster of N fibroblasts, where N = 5 for all results described here. In order to take into account the different sizes of the fibroblast and myocytes we assume that there is only a gap-junctional coupling and no diffusive coupling between myocytes and fibroblasts. Further, we assume that there is no gap-junctional coupling between fibroblasts. The differential equations are solved using the forward Euler scheme, the standard five-point stencil is used for the Laplacian and no-flux conditions are implemented at the edges of the system domain. The value of diffusion constant used is *D* = 0.00154 *cm*^2^/*ms*, and the space- and time- step are 0.25 mm and 0.02 ms respectively. The fibroblasts are distributed randomly as their locations are chosen from a uniform distribution (Supplementary Fig. S1). While most of the results shown here are for one particular distribution with 30% of the lattice points occupied by fibroblasts, we have verified that our results are qualitatively similar for different distributions of the same fraction of fibroblasts. We have also performed simulations where 10% and 20% of the lattice points are filled with fibroblasts. We have used two types of initial conditions generated by two different protocols as suggested in Vandersickel *et al*.^40^. For protocol *P*1, a stimulus of strength 20 *μA/mm*^2^ is applied for a period of 2 ms over a region of size 6 × 200 from a position slightly off center of the simulation domain. In Fig. 1(a–c) we describe this stimulation protocol using a system of size 512 × 512. This protocol mimics the condition that could lead to the origin of arrhythmia via the initial formation of a reentrant wave. Note that for the case of P1 stimulation, the initial condition for the myocytes is obtained by fixing x*G*_*Kr*_ = 1 and y*G*_*CaL*_ = 1 and sending 50 waves at 1 Hz frequency in a 2D tissue without fibroblasts The results shown here are for the dynamics of the single wave in a medium where fibroblasts are interspersed with myocytes having this initial condition. For protocol *P*2, the initial condition is a spiral wave obtained by using *S*1−*S*2 stimulation as shown in Fig. 1(d–f). Stimulus *S*1 is used to set off a plane wave that propagates from one edge of the domain to the other. Once this wave has passed over the half of the domain, the second stimulus *S*2 is applied over the first quarter of the domain thereby producing a wavefront that evolves into a spiral. The aim of this protocol is to study the effect of M-F coupling on the transition from tachycardia (single spiral) to fibrillation (multiple spirals).

## Results

### Single cell coupled to fibroblasts

**Figure 2:**
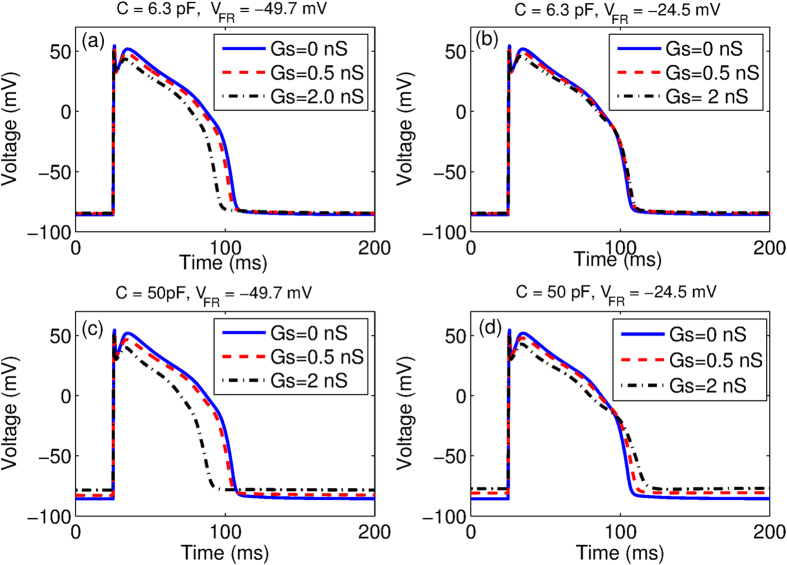
The effect of M-F coupling on single cell action potential, for Gs  = 0 nS (solid line), Gs  = 0.5 nS (broken line) and Gs  = 2.0 nS (dot-dash) for fibroblasts (**a,b**) and myofibroblasts(**c,d**) using resting membrane potentials *V*_*FR*_ = −49.7 mV for (**a** and **c**) *V*_*FR*_ = −24.5 mV for (**b** and **d**). Myocyte parameters used (x*G*_*CaL*_ = 3 and y*G*_*Kr*_ = 0.75) do not produce EADs in the absence of coupling.

**Figure 3:**
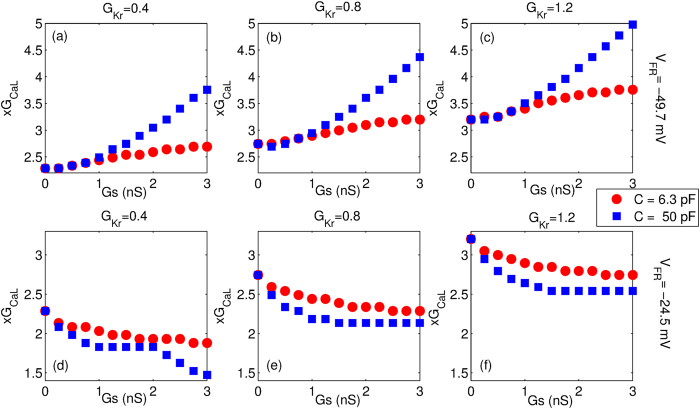
The value of parameter x*GCaL* at which the transition from NO EAD to EAD state occurs is plotted as a function of the strength of M-F coupling for values of y*G*_*Kr*_ = 0.4 (**a,d**), 0.8 (**b,e**) and 1.2 (**c,f**) respectively for C = 6.3 pF (circles) and C = 50 pF (square). Panels (a–c) correspond to resting membrane potential *V*_*FR*_ = −49.7 mV, while panels (d–f) correspond to the case when *V*_*FR*_ = −24.5 mV.

To understand the effect of M-F coupling on myocyte dynamics we first compare the single cell action potential in the presence and absence of coupling as shown in Fig. 2. The parameters are chosen so that in the absence of fibroblast coupling, no EADs are observed. We observe that for the case where *V*_*FR*_ = −49.7 mV, the coupling leads to faster repolarization and hence reduction in action potential duration for both fibroblasts (Fig. 2(a)) and myofibroblasts (Fig. 2(c)). For *V*_*FR*_ = −24.5 mV the M-F coupling leads to a larger increase in APD of the myocyte when coupled to “myofibroblasts” (Fig. 2(d)) than when coupled to “fibroblasts” (Fig. 2(b)). Further, coupling with “myofibroblasts” leads to a greater depolarization of the resting membrane potential especially at large values of *Gs* (Fig. 2(c and d)), than when connected with “fibroblasts” (Fig. 2(a and b)). Supplementary Fig. S2 shows the effect of MF coupling for the case of a myocyte that describes an EAD even in the absence of coupling. In Fig. 3, we plot the value of x *G*_*CaL*_ at which the transition from the NO EAD to EAD state occurs as we vary the strength of the gap-junctional coupling (*Gs*) for y*G*_*Kr*_ = 0.4, 0.8 and 1.2 respectively. Note that *x* and *y* are the multiplicative factors of the maximal conductances for *I*_*CaL*_ and *I*_*Kr*_ respectively. Depending on the resting membrane potential of the fibroblast (*V*_*FR*_), the transition boundary either shifts towards higher (Fig. 3(a–c)) or lower values of *G*_*CaL*_ (Fig. 3(d–f)), with increase in the M-F coupling strength (*Gs*). For a given value *V*_*FR*_, the magnitude of the shift in boundary is larger for the case of coupling with “myofibroblasts” compared to coupling with “fibroblasts”. However the direction of the shift does not depend on whether the myocyte is coupled to “fibroblasts” or “myofibroblasts”. All the possible dynamical states that occur due to the M-F coupling are described using two-parameter phase portraits (see Supplementary Fig. S3).

### Effect of fibroblasts in two-dimensional wave propagation

**Figure 4:**
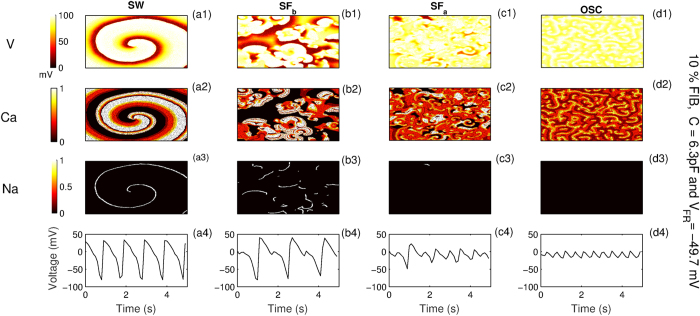
Characterizing the different possible dynamical states and their corresponding time series, due to M-F coupling for stimulation P2. The columns correspond to spiral wave (SW), spiral fibrillation (*SF*_*b*_ and *SF*_*a*_) and oscillatory dynamics respectively. The rows correspond to voltage, state of L-type Ca gates (=*dff*_2_*f*_*Cass*_) and Na gates (=*m*^3^*hj*) respectively. Fully open gates have a value of 1, while fully closed gates have value 0. Parameters used are y*G*_*Kr*_ = 0.2, Gs  = 0.5, with fibroblasts occupying 10% of the randomly chosen lattice points. The values of x (multiples of *G*_*CaL*_) are 2.25, 3.0, 3.5 and 4.75 for SW, *SF*_*a*_, *SF*_*b*_ and OSC states respectively.

We next illustrate the effect of both “fibroblasts” and “myofibroblasts” on wave propagation in a 2D tissue. For this we consider two representative cases namely Fibroblast-1 (fibroblasts) with *C* = 6.3 pF and *V*_*FR*_ = −49.7 mV and Fibroblast-2 (myofibroblasts) with *C* = 50 pF and *V*_*FR*_ = −24.5 mV. Since the effect of coupling in single cells (Fig. 3) is observed to be more pronounced for the case of myofibroblasts (*C* = 50 pF) than for fibroblasts (*C* = 6.3 pF), we expect that the results for myofibroblasts (*C* = 50 pF) with *V*_*FR*_ = −49.7 mV would be more pronounced than for the case with Fibroblast-1 (*C* = 6.3 pF and *V*_*FR*_ = −49.7 mV). Similarly we expect that the results corresponding to the case of fibroblasts (*C* = 6.3 pF) with *V*_*FR*_ = −24.5 mV would be less pronounced than the case of Fibroblast-2 (*C* = 50 pF and *V*_*FR*_ = −24.5 mV). To study the spatial manifestation of the various dynamical states observed for a single cell, we use two different stimulation protocols. For protocol P1 a single wave is initiated from the boundary, while for the second case (P2) a spiral wave is created via the standard S1-S2 protocol. For both cases the dynamics of the wave interacting with fibroblasts is characterized by systematically varying the maximal conductances *G*_*Kr*_ and *G*_*CaL*_. In Fig. 4, we classify the different dynamical states obtained due to myocyte fibroblast interaction for stimulation protocol P2. These are spiral wave (SW), fibrillation mediated via Sodium waves (*SF*_*b*_), fibrillation via Calcium waves (*SF*_*a*_) and phase waves corresponding to the state where the cells continue to oscillate instead of repolarizing (OSC). The columns (a - d) in Fig. 4 correspond to the different dynamical states and rows (1−3) the pseudocolor plots for the voltage and the states of Calcium and Sodium channel gates respectively. The characteristic voltage time series for each dynamical state is shown in row (4).

Below, we briefly describe the features of the dynamical patterns observed for the case of P2, namely *SF*_*a*_, *SF*_*b*_ and OSC. While *SF*_*b*_ and *SF*_*a*_ are characterized by the existence of multiple spirals describing chaotic activity, the dominant ionic currents underlying the two are not the same. The patterns in the case of *SF*_*a*_ are driven by L-type Calcium waves, while Sodium dominates the wave activity in *SF*_*b*_. This is evident from the spatial profile of the ionic gate dynamics and the time series in Fig. 4(b2-b4) (*SF*_*b*_) and Fig. 4(c2-c4) (*SF*_*a*_). For *SF*_*a*_ we find that while there is significant Calcium gate dynamics (Fig. 4(c2)), there is hardly any Sodium gate activity (Fig. 4(c3)). Also the voltage time series (Fig. 4(c4)), shows that the cells do not repolarize completely, an indication that the activity is driven by by Calcium^40^,^43^. The patterns observed during *SF*_*b*_ are driven by Sodium as observed from the spatial profile of the gate dynamics in Fig. 4(b3). Unlike in *SF*_*a*_, the cells repolarize completely (Fig. 4(b4)), indicating active Sodium channel dynamics. Finally, we also observe a state where all the cells are oscillating but because of synchronization seem to generate a wave like activity. Such waves have been identified to be a phase waves^40^, their dynamics being driven by Calcium. For protocol P1 instead of SW we have two other states (not explicitly shown) *viz.*, NO EAD corresponding to the case where wave propagation occurs without oscillations in the action potential, and EAD, the state with the formation of an early afterdepolarization that propagates through the system but does not lead to any sustained electrical activity. For protocol P1, the state characterized by the existence of multiple spirals and excitation fragments (SF) includes both Sodium and Calcium mediated fibrillatory activity i.e., *SF*_*a*_ and *SF*_*b*_. And finally as in protocol P2, OSC corresponds to the case where all cells are oscillating. Here the spatial activity observed is a phase wave and not a wave of excitation.

**Figure 5:**
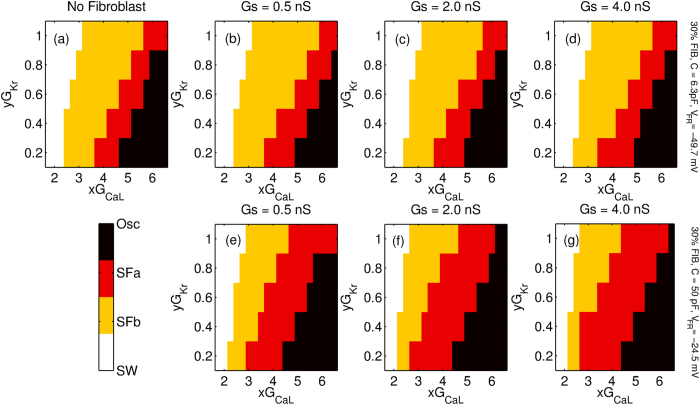
Two-parameter phase diagram of the steady state dynamics for a 2D system with 30% fibroblasts inserted randomly between myocytes and stimulated via protocol P2. The x and y axis correspond to the factor by which *G*_*CaL*_ and *G*_*Kr*_ are multiplied. The phase portraits are shown for three different coupling strengths *Gs* = 0.5 nS, 2.0 nS and 4.0 nS. While (**a**) corresponds to the case with no fibroblasts, (**b–d**) are portraits for Fibroblast-1(C = 6.3 pF, *V*_*FR*_ = −49.7 mV) and (**e–g**) are phase diagrams for Fibroblast-2 (C = 50 *pF* and *V*_*FR*_ = −24.5 mV). The colormap indicates the different dynamical states observed, *viz.* stable rotating spiral wave (*SW*), spiral fibrillation driven by sodium waves (*SF*_*b*_), spiral fibrillation driven by calcium waves (*SF*_*a*_) and fibrillation due to phase waves (OSC).

We first describe the effect of the strength of M-F coupling for protocol P2. In order to study the steady state dynamics we start with a spiral wave for the parameter *xG*_*CaL*_ with *x* = 1.75 and slowly increase the value of x in steps of 0.25 every 4 s. Phase portrait Fig. 5 describes the various dynamical states obtained for different values of M-F coupling conductance (*Gs* = 0.5 nS, 2.0 nS and 4.0 nS) for both Fibroblast-1 (Fig. 5(b–d)) and Fibroblast-2 (Fig. 5(e–g)). For the sake of comparison, we have also plotted the case with no fibroblasts are present (Fig. 5(a)). The phase portrait in Fig. 5 shows that the type of fibroblast coupled to the myocyte determines the nature of the steady state dynamics. M-F coupling with Fibroblast-1 does not significantly change the region of the parameter observed with increasing coupling strength (see Supplementary Fig. S4(a)). On the other hand for the case of Fibroblast-2, we observe a significant shift of the spiral wave (SW) boundary to lower values of *G*_*CaL*_. We further observe that with increased coupling strength, more points turn *SF*_*a*_ or oscillatory, while the region over which we get *SF*_*b*_ dynamics shrinks (see Supplementary Fig. S4).

**Figure 6:**
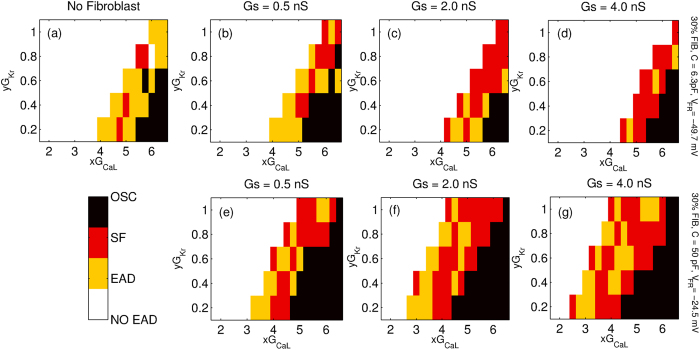
Two-parameter phase diagram of the steady state dynamics for a 2D system with 30% fibroblasts inserted randomly between myocytes and stimulated via protocol P1. The axes correspond to the factors by which *G*_*CaL*_ and *G*_*Kr*_ are multiplied. The phase portraits are shown for three different coupling strengths *Gs* = 0.5 nS, 2.0 nS and 4.0 nS. While (**a**) corresponds to the case with no fibroblasts, (**b–d**) are portraits for Fibroblast-1(C  = 6.3 pF, *V*_*FR*_ = −49.7 mV) while (**e–g**) are phase diagrams for Fibroblast-2 (C  = 50 *pF* and *V*_*FR*_ = −24.5 mV). The colormap indicates the different dynamical states observed, *viz.* wave propagation without any oscillation in action potential (NO EAD), a propagating wave with oscillations in action potential but no persistent activity (EAD), spiral fibrillation consisting of many sources of activation (*SF* includes both *SF*_*b*_ and *SF*_*a*_) and oscillatory fibrillation (OSC).

We next describe the effect of fibroblast coupling on the dynamics of the wave stimulated using protocol P1. Figure 6 describes the various dynamical states obtained for different values of M-F coupling conductance (*Gs* = 0.5 nS, 2.0 nS and 4.0 nS) for both Fibroblast-1 (Fig. 6(b–d)) and Fibroblast-2 (Fig. 6(e–g)). Again for the sake of comparison we have plotted the phase diagram for the case where no fibroblasts are present (Fig. 6(a)). Coupling with fibroblasts significantly alters wave propagation in the medium, the exact dynamics itself depending on the type of fibroblast. We observe that when compared to the case where there are no fibroblasts in the medium, coupling with Fibroblast-1 increases the parameter region over which no EADs or fibrillatory activity are observed. Further, with increase in coupling strength the number of cases that show early afterdepolarization but do not develop arrhythmic activity decreases (see Supplementary Fig. S5(a)). On the other hand, for the case of Fibroblast-2 we observe that the effect of the coupling is reversed, i.e., the M-F coupling promotes arrhythmic dynamics (Fig. 6(e–g)). The coupling induces EAD-mediated fibrillatory patterns even for values of *G*_*CaL*_ and *G*_*Kr*_ that would not have displayed EADs in the absence of coupling.

**Figure 7:**
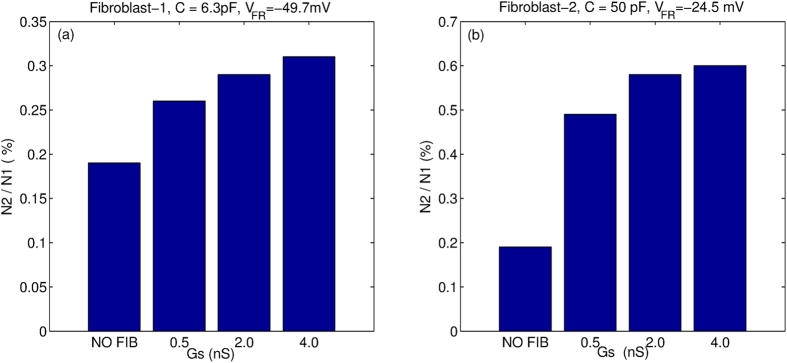
Effect of M-F coupling on the percentage of cases that result in non-trivial pathological dynamics (such as *SF*_*a*_ or *SF*_*b*_ or OSC) as a function of strength of coupling. N1 and N2 are the number of non-trivial cases resulting from protocol P2 and P1 respectively. The results are shown for both Fibroblast-1 (**a**) and Fibroblast-2 (**b**).

We next quantify the effect of M-F coupling in producing non-trivial pathological dynamical states such as spiral fibrillation (*SF*_*a*_ and *SF*_*b*_) and oscillatory dynamics (OSC). For this we consider the ratio of the number of such states observed for both protocols (Fig. 7). N1 is the number of non-trivial pathological dynamical states obtained for stimulation protocol P2, while N2 is the number for protocol P1. We observe that while in general M-F coupling promotes the occurrence of spiral fibrillation or oscillatory dynamics (as measured by the ratio 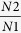), the effect for Fibroblast-2 (Fig. 7 (b)) is almost two times the effect of Fibroblast-1 (Fig. 7 (a)).

**Figure 8:**
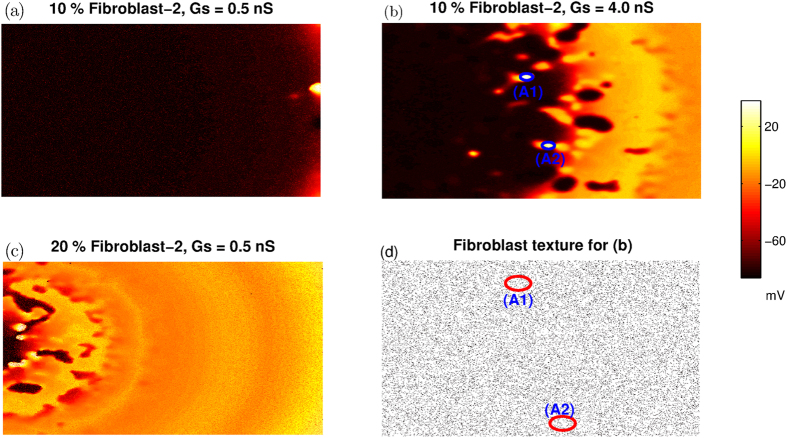
Pseudocolor images of transmembrane potential highlighting the location of initiation of reentrant activity for fibroblast fractions 10% (**a,b**) and 20% (**c,d**). For panels (a,b,d) Fibroblast-2 (C = 50 pF and *V*_*FR*_ = −49.7 mV) is used, while for panel (c) Fibroblast-1 (C = 6.3 pF and *V*_*FR*_ = −24.5 mV) is used.

In order to confirm that the origin of fibrillation like patterns is indeed the effect of M-F coupling, we determine the location where additional waves are first induced for the case of protocol P1. We set y*G*_*Kr*_ = 0.4, and perform the simulations for 20 different values of x*G*_*CaL*_ between 1.75 and 6.5. The results shown in Table. 2 are averaged over 40 simulation runs (two different realisations for each point) for the case of NO FIB. In the case of Fibroblast-1 inserted at 20% of the lattice points, the values shown are averaged over 80 simulation runs (i.e. over four different fibroblast distributions) for both *Gs* = 0.5 nS and *Gs* = 2 nS. For each of the three cases shown in Table. 2 we list the percentage of runs for which no additional depolarizing waves (other than P1) are formed in the medium (col-2), the fibrillatory pattern first occurs at the boundary (col-3) and ectopic activity is initiated away from the boundary (col-4). We observe that for the parameter value chosen here (x*G*_*Kr*_ = 0.4), the fibrillatory waves are mostly initiated at the boundary when fibroblasts are either absent or are weakly coupled (*Gs* = 0.5 nS) to the myocyte. However for larger coupling strengths, ectopic activity is mostly initiated at locations away from the boundary.Note that this table only lists the location of the first occurrence of ectopic activity for each simulation. The location of subsequent occurrences of ectopic activity are not considered. In Fig. 8 we show representative patterns of initiation of spiral wave activity in the presence of fibroblasts. For the case of 10% fibrosis with Fibroblast-2 (*V*_*FR*_ = −24.5 mV and *C* = 50 pF), the reentrant activity (after the initial wave has passed through the medium) occurs at the boundary for small values of coupling (Gs  = 0.5 nS) (Fig. 8(a)). However for stronger coupling (Gs = 4 nS), fibrillatory activity is initiated in the middle of the simulation domain (Fig. 8(b)). We also observe that the location of the initiation of fibrillatory patterns depends on the fraction of fibroblasts present in the medium. While a lattice with 10% fibroblasts (Fibroblast-2) results in initiation at the boundary (Fig. 8 (a)), increasing the fraction to 20% leads to formation of wave activity away from the boundary (Fig. 8(c)). In order to associate the onset of ectopic activity to the underlying fibrotic texture, the spatial distribution of fibroblasts corresponding to Fig. 8(b) is plotted in Fig. 8(d). The circles are the locations in the tissue where ectopic beats first occurred for the case shown in Fig. 8(b). We observe that there is nothing specific about the distribution of fibroblasts at the location where the ectopic activity is initiated, implying that the onset of ectopic beats is not directly related to simple properties of fibrotic texture and needs to be studied further.

## Discussion

In this paper, we have presented results from our numerical study of the effect of gap-junctional coupling between myocytes and fibroblasts on both single cell dynamics and 2D wave patterns. Specifically, we have studied the effect of the fibroblast coupling on EADs and onset of fibrillation by inserting “active” fibroblasts^25^ in a 2D medium of human ventricular cells^36^,^37^. Although the presence of gap-junctional coupling between fibroblasts and myocytes *in vivo* is still debated^14^, there is substantial evidence for the existence of such a coupling *in vitro*^18^,^19^. Further, both cell-culture and simulation studies have shown that such a coupling could have arrhythmogenic effects^32^,^33^,^44^. While these studies have investigated the role of the M-F coupling, they have usually considered fibroblasts to be passive cells that do not express ionic currents. Recent studies have identified ionic currents in fibroblasts that could potentially affect the electrical activity of heart. The key motivation for this paper has been to combine a model of “active” fibroblasts that describes the experimentally observed ionic currents^25^ with a human ventricle model^36^,^37^. We consider two kinds of fibroblasts in our study, namely quiescent fibroblasts and myofibroblasts. Generally the term “fibroblasts” are used for cells in the normal heart, while “myofibroblasts” are used for those occurring in injured hearts^44^. Myofibroblasts are larger in size and have a larger membrane capacitance (*C* = 50 pF) than fibroblasts (*C* = 6.3 pF). We have used the fibroblast capacitance values as suggested in refs 25, 32 and 34. Recent experiments on human atrial fibroblasts suggest a capacitance value ~14.5 pF^45^. For our single cell studies we consider two different resting membrane potential for the fibroblasts (*V*_*FR*_ = −49.7 mV and *V*_*FR*_ = −24.5 mV) and study all four combinations of *V*_*FR*_ and C. For the 2D studies we use two specific combinations of C and *V*_*FR*_, one mimicking a “quiescent fibroblast” (Fibroblast-1) and the other a “myofibroblast” (Fibroblast-2).

We observe in Fig. 2 that the most significant effect of coupling is on the APD and the myocyte resting membrane potential. While coupling with a more negative fibroblast resting membrane potential (*V*_*FR*_ = −49.7 mV) reduces the myocyte APD (Fig. 2 (a and c)), coupling with a fibroblast with less negative resting membrane potential (*V*_*FR*_ = −24.5 mV) increases APD (Fig. 2 (b and d)). On the other hand a larger fibroblast capacitance (*C* = 50 pF) leads to a larger depolarization of resting membrane compared to the case where *C* = 6.3 pF. The primary effect of the M-F coupling is to significantly shift the parameter boundary at which transition to EAD occurs (Fig. 3).We observe that while using a higher fibroblast capacitance of *C* = 14.5 pF as suggested in ref. 45 has a more pronounced effect than using *C* = 6.3 pF, there is no qualitative change in our results (see Supplementary Fig. S6).We observe that *V*_*FR*_ is the critical parameter that determines whether M-F coupling promotes or suppresses EADs. The other parameters such as fibroblast capacitance and strength of the gap-junctional coupling do not critically modify myocyte dynamics but only affect the magnitude by which the transition boundary shifts. The crucial role of *V*_*FR*_ in determining the myocyte dynamics is consistent with both experimental observations^32^ and simulations done using passive cells description of fibroblasts^33^. Supplementary Figs (S7 and S8) show the effect of MF coupling on the ionic currents. The effect of MF coupling is to essentially reactivate the L-type Calcium current. In Fig. S7(d) we observe that there is an increase in late *I*_*CaL*_ similar to what was observed in MacCannell *et al*.^25^. When the resting membrane potential of the fibroblast *V*_*FR*_ = −49.7 mV, this increase in *I*_*CaL*_ is compensated by an increase in the gap-junctional current from myocyte to fibroblast (*I*_*MtoF*_) (Fig S7(c)), resulting in the shortening of the myocyte APD (Fig S7(a)). However when *V*_*FR*_ = −24.5 *mV*, the increase in *I*_*MtoF*_ is not sufficient to compensate the change in late *I*_*CaL*_, resulting in prolongation of the myocyte action potential (Fig S7(a)). Note that here we do not observe EADs both in the absence and presence of coupling. However, for the parameters considered in Supplementary Fig. S8, we observe that while there is no EAD in the absence of fibroblasts, MF coupling promotes EAD. As observed in Fig. S8(b), the main reason for the onset of EAD is the reactivation of *I*_*CaL*_ around *T* = 300 ms. Although there is still an outward current from the myocyte to the fibroblast (*I*_*MtoF*_), it is not sufficient to exceed the value of the reactivated *I*_*CaL*_. This increase in the inward depolarizing current results in the formation of EAD.

We classify the different kinds of 2D patterns observed as a result of the M-F coupling and draw 2D phase portraits. Figures 5 and 6 suggest that the effect of M-F coupling on the promotion or inhibition of EADs and stabilisation or destabilisation of spiral waves depends on the properties of the fibroblast. Thus while coupling with Fibroblast-2 promotes propagating EAD waves (Fig. 6(e–g)) and destabilizes spiral waves (Fig. 5(e–g)), Fibroblast-1 suppresses EADs (Fig. 6(b–d)) and stabilizes spiral waves (Fig. 5(b–d)). However, irrespective of the type of fibroblast, M-F coupling promotes the occurrence of non-trivial pathological dynamics such as spiral fibrillation and oscillatory dynamics (Fig. 7). Thus while M-F coupling with Fibroblast-1 suppresses EAD and stabilizes spiral dynamics, it also gives rise to arrhythmic patterns (SF and OSC) for large values of *G*_*CaL*_. Note that these correspond to parameters that in the absence of fibroblast would have shown a propagating wave of EAD but no spiral fibrillation. Our results do not change qualitatively for other realisations of the fibroblast distribution (for the same number of fibroblasts), but do depend on the percentage of fibroblasts in the medium. For instance, we observe that the effect of the coupling is more pronounced for the medium with 30% fibroblasts than for a medium with 10% fibroblasts.

We find that the location of the initiation of arrhythmic activity depends on the strength of M-F coupling and the fraction of fibroblasts in the medium. For weak coupling (*Gs* = 0.5 nS) and/or small fraction of fibroblasts (10%), fibrillation is initiated at the boundary (Fig. 8(a). On the other hand for stronger coupling (*Gs* = 4.0 nS) and/or larger fraction of fibroblasts (20%), waves originate also from within the medium, away from the boundary (Fig. 8(b,c)). This suggests that the M-F coupling significantly modifies the wave dynamics in the medium, giving rise to new waves of electrical activity far away from the boundary.

The onset of ectopic activity in heterogeneous and fibrotic cardiac tissue was earlier studied in the ORd model for human ventricular cells^33^. However, there are some crucial differences between the earlier study and our work. In Zimik *et al*., the passive fibroblasts (modelled as RC circuits) were randomly attached to the myocytes. Such connections are classified as “single-sided coupling”^14^ where the fibroblasts act as a load that either drains or charges the myocyte. On the other hand, in our work we simulate ‘double-sided coupling’^14^ by inserting the “active” fibroblasts between myocytes in a 2D medium allowing coupling between the fibroblasts and all the non-fibroblast neighbours, thereby creating a heterogeneous medium with spatially varying ionic properties. Furthermore, the mechanisms of EAD generation in the ORd model is substantially different from that in the TNNP model. For example, while EADs are observed at very low pacing frequencies in ORd model, they occur at higher stimulation rates in the TNNP model. In spite of these differences both studies show similar effects for M-F coupling especially with respect to the onset of EADs and easier generation of ectopic beats in fibrotic tissue. Thus the results obtained in our paper are not model specific and are likely to occur in any cardiac cell model that can produce EADs, irrespective of the underlying mechanism.

One potential explanation for the occurrence of such ectopic activity would be to relate their onset to the underlying local fibrotic patterns. Such a pattern could initiate the ectopic activity by reducing the local current sink^31^. However we have not been able to identify any specific local fibrotic patterns that could drive such an ectopic foci, nor have we been able to predict the ectopic sites based on tissue texture (Fig. 7(b,d)). Identifying possible relationships between ectopic sites and the local texture of tissue is extremely important especially in the context of therapy. This is a line of study that needs to be pursued further using statistical approaches and machine learning algorithms^46^ In-silico studies have shown that the presence of EADs can substantially increase the defibrillation threshold in human ventricular tissue^47^. It has also been observed that conduction heterogeneities can help in tissue defibrillation^48^. Since fibrosis introduces spatial heterogeneities it would be interesting to study whether fibrosis can lead to a reduction in the defibrillation threshold in the presence of EADs. It would also be interesting to study the combined effect of fibrosis and heterogeneity as the latter can result in different types of spiral wave dynamics such as drift^49^,^50^. In addition, longer APD associated with EADs can facilitate meandering of the spirals^51^. To summarize, in this *in silico* study we characterize for the first time the different complex dynamics that occur due to gap junctional coupling between myocytes and “active fibroblasts” using models of single cell and 2D human heart tissue.

We conclude by stating the limitations of our study and the scope for future work. We have only studied dynamics in 2D isotropic systems. While fibroblasts themselves have been implicated in increasing anisotropy^52^, the tissue anisotropy itself can promote EADs especially for small values of gap-junctional conductances^31^. Incorporating 3D tissue structure and an anatomically correct description of wave propagation is crucial to understand arrhythmogenesis in the actual heart^53^. However in this paper we have only focussed on characterizing the effect of M-F coupling on the manifestaton of complex wave patterns in 2D domain by performing two-parameter studies for two kinds of fibroblasts. Studying the manifestation of EADs due to M-F coupling in 3D domains will be an area of future work. Experimental data suggest that fibroblasts may function as mechano-electric transducers^54^. In this context, it will be of interest to study the effect of mechano-sensitivity of fibrotic tissue on generation of EADs using methods suggested in Keldermann *et al*.^55^. Since the effect of fibroblast coupling is possibly dependent on the type of action potential^24^, it would be of interest to compare our results with other models of human heart tissue. Since the focus of our study was to showcase the different dynamical states that fibroblast-myocyte coupling can give rise to, we fixed the number of fibroblasts attached to a myocyte to be 5 in all our simulations. But the number of fibroblasts coupled to a myocyte is known to be an important factor that affects the action potential^25^ and can be systematically varied to investigate its effect of on wave propagation, a topic for future study.

## Additional Information

**How to cite this article**: Sridhar, S. *et al*. Effect of myocyte-fibroblast coupling on the onset of pathological dynamics in a model of ventricular tissue. *Sci. Rep.* **7**, 40985; doi: 10.1038/srep40985 (2017).

**Publisher's note:** Springer Nature remains neutral with regard to jurisdictional claims in published maps and institutional affiliations.

## Supplementary Material

Supplementary Material

## Figures and Tables

**Table 1:** In this table, we list the values for the fibroblast parameters, *viz.*, capacitance (C), resting membrane potential (*V*_*FR*_) and coupling strength (Gs) used for the results described in the paper.

Dimension	C (pF)	*V*_*FR*_ (mV)	Gs (nS)
0D	6.3, 50.0	−49.7, −24.5	0–3
2D	6.3, 50.0	−49.7, −24.5	0.5, 2.0, 4.0

**Table 2:** In this table, we list the percentage of cases for the initial location at which additional waves first occur for the case of protocol P1.

Case	No new waves (%)	First new wave from boundary (%)	First new wave from middle (%)
NO FIB	95	5	0
FIB-1, Gs = 0.5 nS	90	10	0
FIB-1, Gs = 2.0 nS	82.5	2.5	15

Rows correspond to different cases considered, *viz.*, where there are no fibroblasts (row-1), for the case of 20% Fibroblast-1 (*V*_*FR*_ = −49.7 mV and *C* = 6.3 pF) with *Gs* = 0.5 nS (row-2) and *Gs* = 2.0 nS (row-3). Simulations are performed by fixing y*G*_*K*_*r* = 0.4 and varying x*G*_*CaL*_ values between 1.75 and 6.5 in steps of 0.25. The percentages are calculated over 40 (NO FIB) and 80 (Fibroblast-1) trials respectively.
