# Supplementary material for: Effect of myocyte-fibroblast coupling on the onset of pathological dynamics in a model of ventricular tissue
Source: Sci Rep. 2017 Jan 20;7:40985. doi: 10.1038/srep40985 (PMC5247688; doi:10.1038/srep40985)
Supplement: Supplementary Material [file srep40985-s1.pdf]

SUPPLEMENTARY MATERIAL FOR
Effect of myocyte-fibroblast coupling on the onset of pathological dynamics in a model of ventricular tissue.

S. Sridhar, N. Vandersickel and A. V. Panfilov

Contents

1. Fig S1: Fibroblasts are inserted randomly between myocytes on a system of size 1024×1024. 10 percent of the lattice points are occupied by fibroblasts (black squares) while the remaining points are occupied by myocytes. (b) Schematic diagram illustrating diffuse fibrosis with a lattice of myocytes (circles) interspersed with fibroblasts (ellipses) coupled to the each other via gap-junctions (broken lines).

2. Fig S2: The effect of coupling with Fibroblast-1 (a) and Fibroblast-2(b) on a single cell action potential for x$G_{CaL} = 5.0$ and y$G_{Kr} = 0.4$ for the case of no coupling (solid line), $Gs = 0.5$ nS (broken line) and $Gs = 2.0$ (dot-dash line).

3. Fig S3: Phase diagram for the different forms of action potentials in the xG_{CaL}-yG_{Kr} parameter space for the case of one myocyte coupled to 5 fibroblasts as a function of strength of M-F coupling (Gs), with x and y corresponding to the factor by which the maximal conductance of I_{Kr} and I_{CaL} are multiplied. The top row (a-c) correspond to the case with fibroblast parameters ($V_{FR} = -49.7$ mV and $C = 6.3$ pF) while the bottom row (d-f) corresponds to the case with parameters ($V_{FsR} = -24.5$ mV and $C = 50$ pF). Cases that do not describe any reversal of the action potential before complete repolarization are represented as NO EAD, while those that show such a reversal are denoted EAD. OSC corresponds to action potentials that oscillate without returning to their resting state.

4. Fig S4: Fraction of simulations leading to the different dynamical states observed in Fig.5 for both Fibroblast-1 (a) and Fibroblast-2 (b) are shown as a function of the M-F coupling strength (Gs). For ease of comparison the results for the case without fibroblasts is also plotted.

5. Fig S5: Fraction of simulations leading to the different dynamical states observed in Fig.6 for both Fibroblast-1 (a) and Fibroblast-2 (b) are shown as a function of the M-F coupling strength (Gs). For the sake of comparison the results for the case without fibroblasts is also plotted.

6. Fig S6: The value of parameter xG_{CaL} at which the transition from NO EAD to EAD state occurs is plotted as a function of the strength of M-F coupling for values of y$G_{Kr} = 0.4$ (a, d), 0.8 (b, e) and 1.2 (c, f) respectively for the case $C = 6.3$ pF (circle), $C = 14.5$ pF (diamond) and $C = 50$ pF (square). Panels (a, b, c) correspond to resting membrane potential $V_{FR} = 49.7$ mV, while panels (d, e, f) correspond to the case when $V_{FR} = 24.5$ mV.

7. Fig S7:Illustration of the effect of MF coupling on action potentials of myocyte (a) and fibroblast (b), current flowing from a myocyte to ($N = 5$) coupled fibroblasts I_{MtoF} (c) and myocyte L-type calcium current (d) for the cases of MF coupling to zero fibroblast (solid line), 5 fibroblasts with $V_{FR} = -49.7mV$ (broken line) and $V_{FR} = -24.5mV$ (dot-dash line). Fibroblast capacitance $C_F = 50$ pF and strength of MF coupling is $Gs = 1nS$. The current flowing between myocyte and fibroblasts is $I_{MtoF} = -N \times \frac{Gs}{C_m} \times (V_{Myo} - V_F)$, where V_{Myo} and V_F are myocyte and fibroblast voltages respectively and $C_m = 185$ pF is the myocyte membrane capacitance. For the parameters used here (x$G_{CaL} = 2.0$ and y$G_{Kr} = 0.4$) while no EADs are observed even in the presence of coupling, depending on the value of V_{FR} the myocyte action potential duration either shortens (broken line) or lengthens (dot-dash line).

8. Fig S8: Illustration of the effect of MF coupling on action potentials of myocyte and fibroblast (a), myocyte L-type calcium current (b) and the current flowing from a myocyte to ($N = 5$) coupled fibroblasts I_{MtoF} (c) for the cases of MF coupling with zero fibroblast (solid line), 5 fibroblasts with $V_{FR} = -49.7mV$ (broken line) and $V_{FR} = -24.5mV$ (dot-dash line). Fibroblast capacitance $C_F = 50$ pF and strength of MF coupling is $Gs = 1nS$. The current flowing between myocyte and fibroblasts is $I_{MtoF} = -N \times \frac{Gs}{C_m} \times (V_{Myo} - V_F)$, where V_{Myo} and V_F are myocyte and fibroblast voltages respectively and $C_m = 185$ pF is the myocyte membrane capacitance. For the parameters used here (x$G_{CaL} = 3.0$ and y$G_{Kr} = 0.4$), EAD occurs only in the presence of MF coupling (broken line).

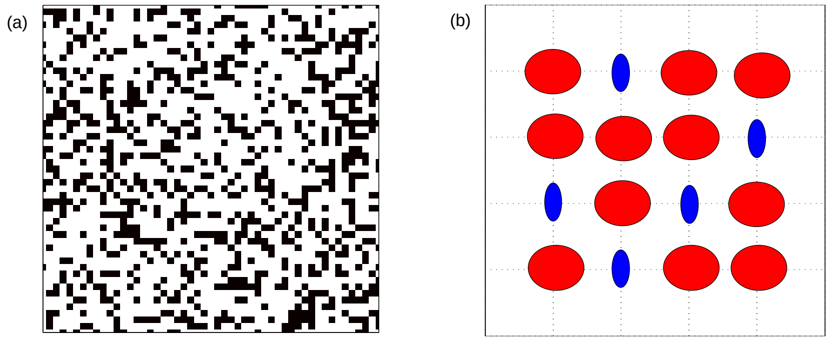

Figure 1: (a)Fibroblasts are inserted randomly between myocytes on a system of size 1024×1024. 10 percent of the lattice points are occupied by fibroblasts (black squares) while the remaining points are occupied by myocytes. (b) Schematic diagram illustrating diffuse fibrosis with a lattice of myocytes (circles) interspersed with fibroblasts (ellipses) coupled to the each other via gap-junctions (broken lines).

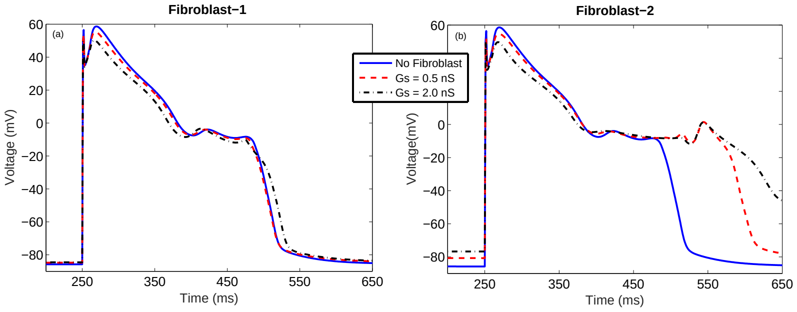

Figure 2: The effect of coupling with Fibroblast-1 (a) and Fibroblast-2(b) on a single cell action potential for xG_{CaL} = 5.0 and yG_{Kr} = 0.4 for the case of no coupling (solid line), $Gs = 0.5$ nS (broken line) and $Gs = 2.0$ (dot-dash line).

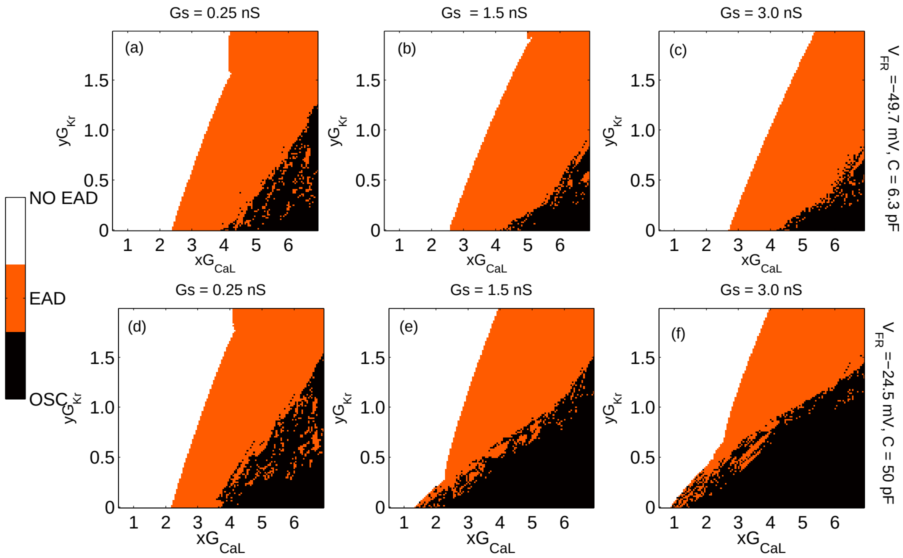

Figure 3: Phase diagram for the different forms of action potentials in the xG_{CaL}-yG_{Kr} parameter space for the case of one myocyte coupled to 5 fibroblasts as a function of strength of M-F coupling (Gs), with x and y corresponding to the factor by which the maximal conductance of I_{Kr} and I_{CaL} are multiplied. The top row (a-c) correspond to the case with fibroblast parameters ($V_{FR} = -49.7$ mV and $C = 6.3$ pF) while the bottom row (d-f) corresponds to the case with parameters ($V_{FsR} = -24.5$ mV and $C = 50$ pF). Cases that do not describe any reversal of the action potential before complete repolarization are represented as NO EAD, while those that show such a reversal are denoted EAD. OSC corresponds to action potentials that oscillate without returning to their resting state.

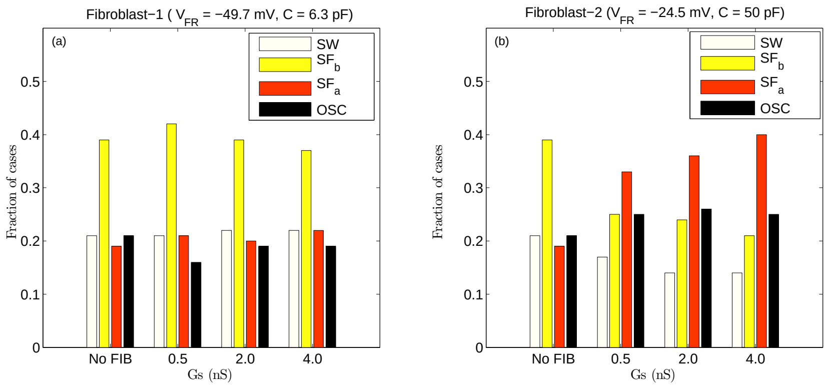

Figure 4: Fraction of simulations leading to the different dynamical states observed in Fig.5 for both Fibroblast-1 (a) and Fibroblast-2 (b) are shown as a function of the M-F coupling strength (Gs). For ease of comparison the results for the case without fibroblasts is also plotted.

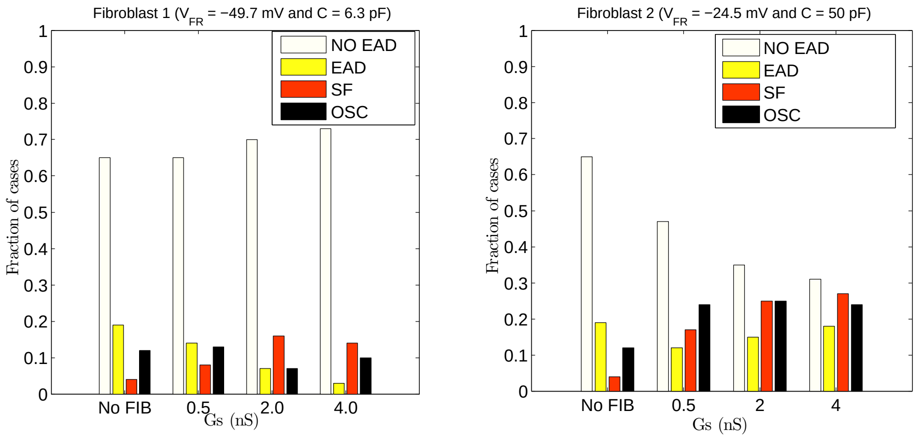

Figure 5: Fraction of simulations leading to the different dynamical states observed in Fig.6 for both Fibroblast-1 (a) and Fibroblast-2 (b) are shown as a function of the M-F coupling strength (Gs). For the sake of comparison the results for the case without fibroblasts is also plotted.

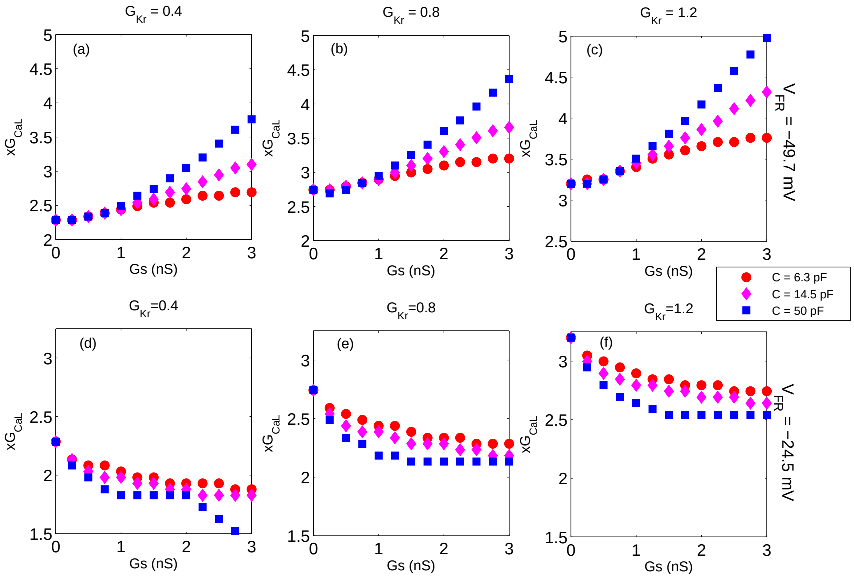

Figure 6: The value of parameter xG_{CaL} at which the transition from NO EAD to EAD state occurs is plotted as a function of the strength of M-F coupling for values of yG_{Kr} = 0.4 (a, d), 0.8 (b, e) and 1.2 (c, f) respectively for the case $C = 6.3$ pF (circle), $C = 14.5$ pF (diamond) and $C = 50$ pF (square). Panels (a, b, c) correspond to resting membrane potential $V_{FR} = 49.7$ mV, while panels (d, e, f) correspond to the case when $V_{FR} = 24.5$ mV.

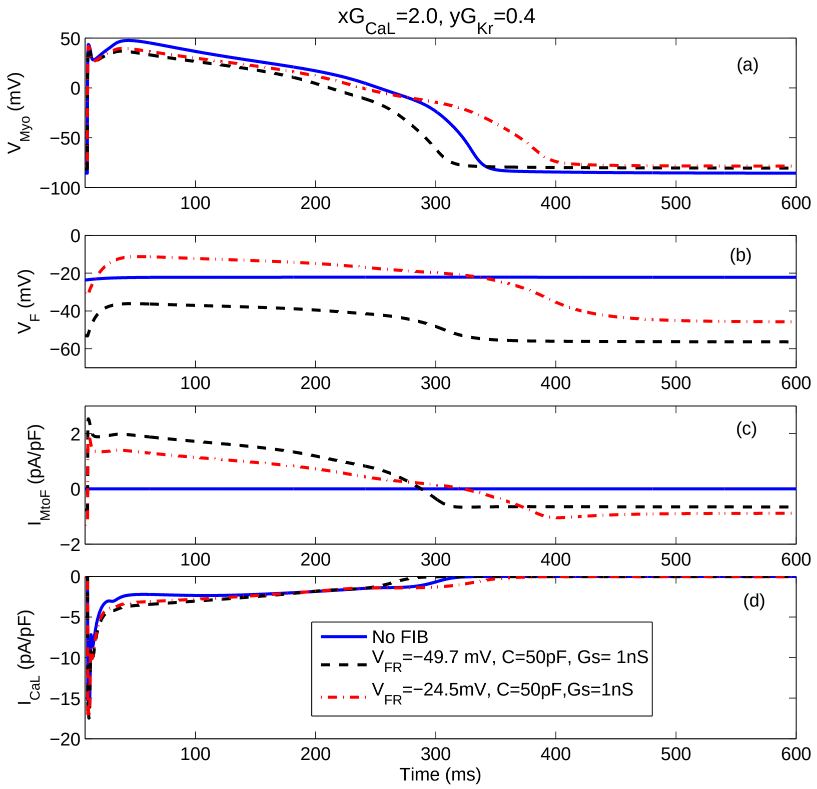

Figure 7: Illustration of the effect of MF coupling on action potentials of myocyte (a) and fibroblast (b), current flowing from a myocyte to ($N = 5$) coupled fibroblasts I_{MtoF} (c) and myocyte L-type calcium current (d) for the cases of MF coupling to zero fibroblast (solid line), 5 fibroblasts with $V_{FR} = -49.7mV$ (broken line) and $V_{FR} = -24.5mV$ (dot-dash line). Fibroblast capacitance $C_F = 50$ pF and strength of MF coupling is $Gs = 1nS$. The current flowing between myocyte and fibroblasts is $I_{MtoF} = -N \times \frac{Gs}{C_m} \times (V_{Myo} - V_F)$, where V_{Myo} and V_F are myocyte and fibroblast voltages respectively and $C_m = 185$ pF is the myocyte membrane capacitance. For the parameters used here (x$G_{CaL} = 2.0$ and y$G_{Kr} = 0.4$) while no EADs are observed even in the presence of coupling, depending on the value of V_{FR} the myocyte action potential duration either shortens (broken line) or lengthens (dot-dash line).

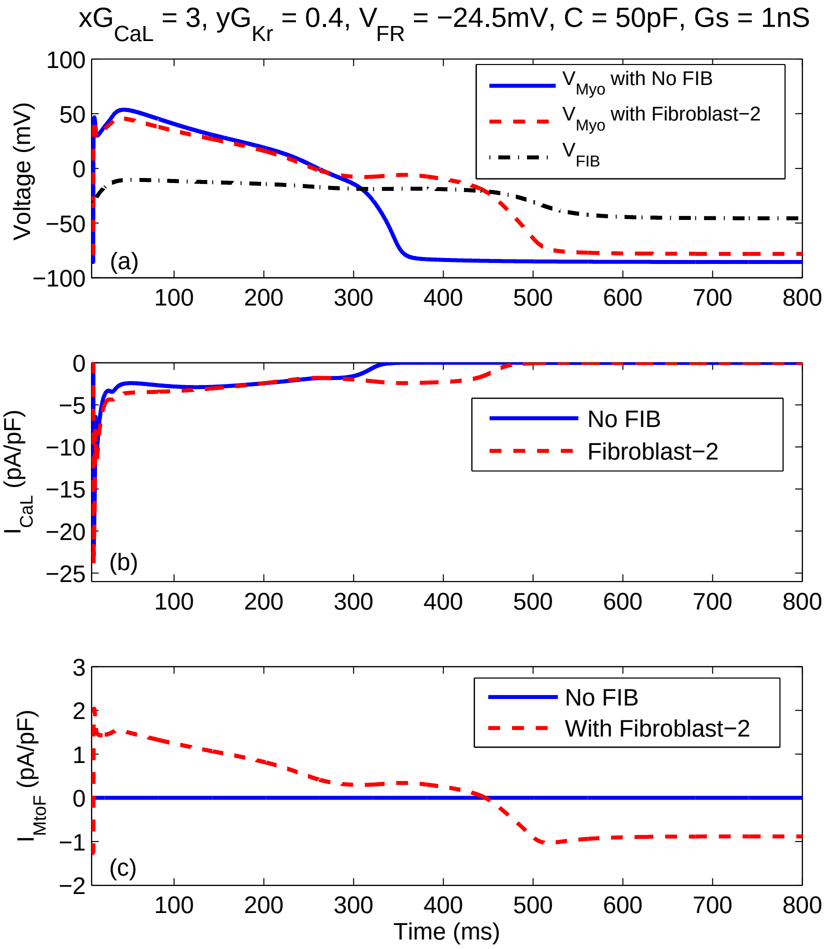

Figure 8: Illustration of the effect of MF coupling on action potentials of myocyte and fibroblast (a), myocyte L-type calcium current (b) and the current flowing from a myocyte to ($N = 5$) coupled fibroblasts I_{MtoF} (c) for the cases of MF coupling with zero fibroblast (solid line), 5 fibroblasts with $V_{FR} = -49.7mV$ (broken line) and $V_{FR} = -24.5mV$ (dot-dash line). Fibroblast capacitance $C_F = 50$ pF and strength of MF coupling is $Gs = 1nS$. The current flowing between myocyte and fibroblasts is $I_{MtoF} = -N \times \frac{Gs}{C_m} \times (V_{Myo} - V_F)$, where V_{Myo} and V_F are myocyte and fibroblast voltages respectively and $C_m = 185$ pF is the myocyte membrane capacitance. For the parameters used here (x$G_{CaL} = 3.0$ and y$G_{Kr} = 0.4$), EAD occurs only in the presence of MF coupling (broken line).
